# The Power of Shared Embodiment: Renegotiating Non/belonging and In/exclusion in an Ephemeral Community of Care

**DOI:** 10.1007/s11013-020-09675-5

**Published:** 2020-04-19

**Authors:** Anita von Poser, Edda Willamowski

**Affiliations:** 1 Institute of Social and Cultural Anthropology and Collaborative Research Center 1171 Affective Societies: Dynamics of Social Coexistence in Mobile Worlds, Freie Universität Berlin, Landoltweg 11, 14195 Berlin, Germany; 2 Collaborative Research Center 1171 Affective Societies: Dynamics of Social Coexistence in Mobile Worlds, Freie Universität Berlin, Habelschwerdter Allee 45, 14195 Berlin, Germany

**Keywords:** Affective community, Embodiment, Belonging, Care, Emplaced ethnography

## Abstract

In this article, we explore the power of shared embodiment for the constitution of an affective community. More specifically, we examine how people afflicted by long-term, arduous experiences of war, migration, and discrimination sensually articulate and, at least temporarily, renegotiate feelings of non/belonging, care, and in/exclusion. Methodologically, we draw on emplaced ethnography and systematic phenomenological go-alongs with a group of elderly migrants, born and raised in different parts of Vietnam, who had arrived in Germany within different legal–political frameworks and who, during the time of our psychological–anthropological research, frequented the same psychotherapeutic clinic. We apply the notion of “affective communities” (Zink in Affective Societies: Key Concepts. Routledge, New York, 2019) to grasp how the group experienced a sensual place of mutual belonging outside the clinic when moving through different public spaces in Berlin as part of their therapy. Particular attention is paid to the participants’ embodied and emplaced memories that were reactivated during these excursions. Shared sensations and spatiality, we argue, made them feel they belonged to an ephemeral community of care that was otherwise hardly imaginable due to their distinct individual biographies, contrasting political attitudes, and ties to different social collectives. In analyzing this affective community, we highlight how significant spatio-sensorial modes of temporal solidification can be in eliciting embodied knowledge that positively contributes to therapeutic processes.

## Introduction

On a grey, cloudy, and windy day in late March 2017, we organized a boat cruise (a so-called “historical sightseeing cruise”) on the Spree river for the participants of a bi-weekly group therapy for Vietnamese migrants that usually took place within a psychotherapeutic outpatient clinic in Berlin.^1^ For many years, the participants, all of them elderly Vietnamese-born women and men, had been suffering from unvoiced feelings of non-belonging and social exclusion. These feelings were directly linked to their often arduous migratory experiences, which could not only be similar but also quite diverse, depending on their respective biographies, their contrasting political attitudes, and their ties to different social collectives. The boat cruise was part of one of four systematic phenomenological go-alongs which we undertook with the participants outside of the clinic as part of an applied and engaged anthropological–psychiatric research framework (Heyken et al. 2019; Nguyen et al. in print). Shortly before the boat was to depart, we realized that Mr. Th,^2^ one of the male participants of the group, was missing. Mr. B, another participant, offered to call Mr. Th to ask about his whereabouts. After a few minutes, however, Mr. Th arrived. Upon the others’ inquiries of why he was late, Mr. Th spontaneously raised his arms and playfully excused his delay with the words “hey, we are all sick!” He humorously added that he had been waiting for Mr. B at an agreed place nearby, which the latter had obviously forgotten, and that this was the reason for his delay. In a similarly humorous way, all the others who were present on that day commented on the situation referring to their statuses as “outpatients of a psychotherapeutic clinic”, and were joined in laughter and an intense affective resonance.

Over the course of this article, we aim to show that this short vignette marks one salient and consolidating moment of what this specific group came to experience as an ephemeral community of care. Despite its ephemerality, the affective intensity of this experience gave the participants new orientations for considering their selves, lives, and surroundings and for reframing their feelings.

In the following, we will first introduce the notion of “affective communities” (Zink 2019), based on which we argue that shared sensations and spatiality allowed our participants to renegotiate feelings of non/belonging, care, and in/exclusion. We will then briefly explain our specific methodological choices before we analyze how shared, embodied as well as emplaced memories (Pink 2015) evoked specific spatio-sensorial ways to temporarily tie the participants together as an affective community. Finally, we will address the value of eliciting embodied and often ‘hidden’ knowledge for therapeutic processes.

## Affective Communities and Renegotiations of Non/belonging and In/exclusion

Despite different reasons for frequenting the psychotherapeutic clinic, there were at least three common aspects that united the elderly participants. First, they had all to different degrees lost their capacity to relate and resonate with others in ways which made them feel safe, included, and properly cared for by others, as well as their capacity to care for others on emotionally recognized terms. Second, they had felt incapable of openly addressing this loss over many years within and beyond their circle of peers. Due to severe afflictions and increasing social conflicts, the participants had either gradually or abruptly withdrawn from society, depending on their individual affective lives. In speaking of “affective lives” (von Poser 2018) from a psychological–anthropological lens, we frame the emotional experiences in people’s life-courses as the dynamic and open outcome of complex, and sometimes arduous, affective processes of dealing with felt differences and of doing and undoing belonging over the entire course of life (cf. Röttger-Rössler 2018). Some had withdrawn because of acute distress, others had done so because of accumulated distress. Nevertheless, all expressed the feeling of being numb, powerless, and dependent. Third, as a consequence of this withdrawal, they all felt incapable not only of maintaining feelings of belonging but also of “[s]ensing and making sense of […] feelings of estrangement [and] discomfort” (Mattes et al. 2019:302).

In other words, they could no longer rely on their “emotion repertoires”, by which we mean the shared frames of reference which are internalized during processes of socialization and which enable individuals and collectives to encode and decode felt experiences as discrete emotions on the levels of discourse, practice, and embodiment (von Poser et al. 2019:243). As a result, they were emotionally overwhelmed by felt differences with detrimental effects on their level of affect.^3^ To give one example of such a felt difference: a male patient told Edda of the pressure he always felt before meeting peers. Before his afflictions had grown in severity, he had, for instance, enjoyed extended visits by his peers at his home. Such visits were usually accompanied by convivial activities such as sharing a meal or taking sight-seeing trips through the city. Moreover, inviting visitors socially and emotionally augmented his status as the head of the family, as it allowed him to perform responsibility in normative terms. With his increasing distress, he hardly felt capable of bearing such encounters any longer. His normative understanding of how to properly feel and behave in such encounters now collided with his actual withdrawal.

Affective dynamics are part and parcel of feelings of belonging. As the editors to this special issue suggest “to belong […] is to share values, networks, and practices and importantly to feel accepted and recognized as a morally equivalent member of a community.” Thus, as the example above indicates, we learned that the participants’ social and spatio-sensorial senses of belonging had been strongly mitigated, which in turn negatively impinged upon their capacities to live sociable lives within society. Put differently, they felt socially displaced and were no longer part of “affective communities,” understood here as the spaces that afford actors the possibility to establish (new) common ground or an “educated social sensibility” (Zink 2019:291) with regard to the recognition and negotiation of affects and emotions. Following cultural sociologist Veronika Zink,

“the concept of affective communities depicts a specific form of collectivity that can be characterized by a shared sensuality eliciting an implicit sense of commonality and immediateness (…). Affective communities are momentary connections of social immediacy that are driven by ‘the impulse of sociability’ (…), that is, of a playful form of practicing convivial connectivity” (Zink 2019:289–290).

Zink explores a phenomenologically inspired notion of relational affects in order to point to the significance of shared, embodied experiences “between affected and affecting social bodies” (2019:289) for the temporal and transient solidarization and de-solidarization that she considers indicative of affective communities. While building on this notion, we simultaneaously expand it by combining the affective communities framework with “embodied” *and* “emplaced ethnography.” Following Sarah Pink, we do not just make a plea for “the integration of mind and body” as posited by embodiment theories but apply an “emplaced ethnography that attends to the question of experience by accounting for the relationships between bodies, minds, and the materiality and sensoriality of the environment” (2015:28). A focus on “emplacement,” then, needs a corresponding methodology in motion, to which we will turn later.

Zink’s notion of affective communities is productive for us as well because of the linkage she makes to process-oriented thinking in social theory (e.g. Elias 2000): societies per se are processes, they are formed and deformed, gain or lose stability, because they are made, unmade, and remade within continuously consolidating and dissolving affective communities. In relating her understanding of affective communities to contemporary social theories that posit “the social as a process in the making” (2019:296), thus rejecting classical social theories of the modern, rationally ordered, static, and indissoluble society, Zink considers affective communities to be “the modus operandi of contemporary affective societies” (2019:297). In other words, affective communities are integral parts of societies because they fundamentally shape their dynamics.

The particular affective community that we wish to address as an ephemeral community of care only gradually emerged over a series of encounters in distinct “affective niches” (Colombetti, Krueger and Roberts 2018:1) in and beyond the clinic in which the participants (re-)activated implicit embodied knowledge through shared sensations and spatiality.^4^ In this process, they also re-negotiated facets of attentiveness, responsiveness, competence, and responsibility, which are important dimensions of a relational understanding of care (Tronto 1993; Sevenhuijsen 2003; von Poser 2017). Their first encounters in the clinic, in fact, were marked by mutual mistrust due to their individual biographies, contrasting political attitudes, and ties to different social collectives (Heyken et al. 2019), aspects we will explore in greater detail below. Moreover, all participants were hesitant to reveal their distinct afflictions in front of each other because of the fear of stigmatization and ‘loss of face’ (Nguyen et al. in print), which, according to paramount Confucian values with which they had grown up in Vietnam, has significant social and moral implications in relation to people’s statuses and feelings of pride or shame. Therefore, the therapist invited the participants to expose intimate personal details during single therapy sessions. During the group therapy sessions, in turn, they could relationally expose the very fact *that* they felt estranged because of their afflictions and that a particular and previously unrecognized type of care was necessary to deal with their respective states of being. Care, after all, involves

“*processes of creating*, *sustaining and reproducing bodies*, *selves and social relationships* (…). They encompass practices, politics and discourses undertaken by individuals and social institutions, immersed in diverse relations of power” (Nguyen, Zavoretti and Tronto 2017:202; italics in original).

Always tied to social, cultural, historical, and political dynamics, care as both a processual and analytical concept takes into account the different, yet interrelated dimensions of “‘caring for the self’, ‘caring for the others’ and ‘caring for the world’, while accommodating both normative and critical interpretations of care” (Nguyen, Zavoretti and Tronto 2017:202).

With this notion of care in mind, we argue that the participants gradually restored their sense of their bodies, selves, and social relationships. Practically speaking, they gained common ground during particular sessions in which their senses were triggered. Rather than asking how they had arrived in Germany, what they had experienced, what they had lost or left behind or what each of them had achieved, our anthropological–psychiatric team asked them to evaluate specific textures, smells, sounds, and visual sceneries. Thus, a sphere was created in which a “reciprocal mode of sensual affections [..] unresistingly and transversally move[d] through bodies and bec[ame] intensified and amplified by them” (Zink 2019:293). In stressing “the social power of sensuality” (Zink 2019:291), a psychoeducational process was set in motion which encouraged the participants to relate their individual states of being to shared bodily practices and perceptions. This synchronization of sensual experiences led to an intensified affective resonance through which the mutual mistrust that we had initially observed started to dissolve.

Noticing that an affective community was about to evolve in the ‘safe space’ of the clinic, we wondered from a decidedly phenomenological–anthropological perspective how this process would further unfold in worlds and emplaced situations of life outside the clinic, which the participants broadly perceived as spaces of social conflicts, structural disadvantages, and misunderstandings, and from which they had withdrawn.

## Phenomenological Go-alongs Outside the Clinic

In view of today’s often fragmented and discontinuous contexts of care, we saw the need to apply a particular methodology in order to be able to better comprehend these contexts. Our phenomenological “go-alongs” (Kusenbach 2003), moreover, were pursued as part of our joint ethnographic fieldwork within a larger interdisciplinary and engaged collaboration embracing sociocultural anthropologists (us), cultural psychiatrists and psychologists, and social workers.

In conducting our anthropological excursions as phenomenological go-alongs, we were interested in the “body as a site of knowing” (Pink 2015:27; cf. Desjarlais and Throop 2011) and therefore wished to focus on the participants’ immediate sensorial perceptions, embodied and emplaced understandings, and affective appropriations of spatio-social worlds. In paying attention to how social actors “move through, and interact with, their physical and social environment,” we intended to observe “spatial practices in situ while accessing their experiences and interpretations at the same time” (Kusenbach 2003:463). The go-along method is in line with sensory ethnography (Pink 2015) and with phenomenological approaches especially “in affect studies that emphasize the situated embodiment of affect and the affective sensitivity of situated bodies” (Slaby and Mühlhoff 2019:34). We not only sought to grasp how spatio-social settings evoke feelings and memories, but also intended to get a sense of the everyday practical and deeply embodied

“aspects of human experience that tend to remain hidden to observers and participants alike. They make visible and intelligible how everyday experience transcends the here and now, as people weave previous knowledge and biography into immediate situated action” (Kusenbach 2003:478).

The question guiding our endeavor was whether such embodied knowledge could be retrieved as a hidden resource to contrast, or even mitigate, felt struggles linked to non-belonging and exclusion. Eventually, the ethnographic insights that we gained during the anthropological excursions were complemented with the insights from our collaborating colleagues in the clinic (Heyken et al. 2019).

Methodologically, we started with silent participatory observations in the waiting room of the outpatient clinic. From here, we followed the participants over the course of a two-year cognitive behavior group therapy within the clinic with a focus on affects and emotions under the guidance of the psychiatrists of our interdisciplinary team (Nguyen et al. in print; von Poser et al. 2019). Apart from that, nine participants agreed to move about as a group in the city of Berlin during recreational excursions lasting three to five hours, which we, the anthropologists, organized together with our team’s psychologist.

Prior to each excursion, we handed out basic information in Vietnamese about the locations that we wished to visit and asked the participants for their opinions. The first excursion in March 2017 encompassed a boat cruise on the Spree river, a visit to the *Tränenpalast* (‘Palace of Tears’), a former border crossing point between East and West Germany which was turned into a documentation center reminding visitors of Germany’s partition, and the visit of a traditional German café. Our second excursion in June 2017 led us to Berlin’s *Botanical Garden* where we did “mindful movements”^5^ under the guidance of the psychologist. We also asked the participants to engage in a photovoice exercise as a means of narrating visualized feelings by “taking photos that show or symbolize what triggers emotions in them” (Röttger-Rössler, Scheidecker and Lam 2019:78). Upon their repeated suggestions that we should also eat together, the day ended with a joint picnic. Due to the seemingly overall positive experiences in the natural surroundings of the *Botanical Garden*, we decided to take our third excursion in September 2017 to the *Pfaueninsel* (‘Peacock Island’), a well-known, tiny, and picturesque island in the Havel river in the southwest of Berlin. On our last excursion, which took place almost a year later in August 2018, we planned to visit another public park. Due to bad weather, however, we changed plans and visited another museum depicting *Everyday Life in Communist East Germany*. Again, we were asked to end the day with a shared meal, this time in a Pan-Asian restaurant that one participant recommended.

The participants, four women and five men, first met each other in the clinic in 2016. They all had afflictions stemming from long-term arduous experiences of war, migration, and discrimination but were diverse with regard to social status, age, and migratory history; the youngest being around 50, the oldest around 70 years of age. As mentioned earlier, in the beginning of the therapy most of them faced each other with mistrust resulting from different legal–political circumstances or “regimes of mobility” (Glick Schiller and Salazar 2013), which had impacted their departures from Vietnam and arrivals in Germany differently. In fact, “[b]oth Germany and Vietnam were countries divided by war whose national divisions remained long after the battlefields fell silent” (Behrens and Đĩnh 2019:89). One woman and two men, we will call them Mrs. L, Mr. S, and Mr. B, shared the experience of having escaped from the South of Vietnam in the aftermath of the socialist seizure of power in 1975 and having arrived as humanitarian refugees in former West Berlin. Each of them was suffering from the long-term consequences of discrimination, forced migration, and a deep sorrow linked to loss of and longing for their lost homes. Mrs. T and Mrs. Q, as well as Mr. Th, Mr. C, and Mr. A, in contrast, had arrived in the former German Democratic Republic (GDR) as contract workers and had stayed after Germany’s reunification under insecure legal conditions. They had all lost their residence statuses and workplaces and had to survive in precarity for several years. Some still live in this limbo. Also, they faced racism as well as the pressure of meeting the expectations to transnationally support their families in Vietnam. Only one woman, Mrs. N, had arrived by means of family reunification by the end of the 1990s to live with her child’s family. In caring for her grandchildren, she performed a familiar role within her family. Still, she felt out of place because she herself felt lonely and neglected by her children who had to work hard day by day.

As we will highlight in the following section, a phenomenological approach with a focus on shared sensorial and emplaced perceptions was an important step to bring to the fore hidden embodied knowledge that re-enabled the participants to relate and resonate in meaningful ways. Importantly, this approach allowed for the articulation of felt dissonances that this particular generation would normally keep to themselves according to deeply engrained Confucian values linked to gender, age, and hierarchy.

## An Ephemeral Community of Care

In this part, we address the ways in which shared sensations and spatiality gradually led to the formation of an ephemeral community of care. We do so by exploring joint movements, the sharing of foodways and the shared sensing of plants, as well as shared and visualized interpretations of sensually stimulating moods.

### Shared Spatial Explorations

The aim of our first excursion was to provide a space in which the participants could exchange their distinct narratives of migration without reproducing political animosities that discursively still prevail in stereotypical ways among elderly Vietnamese in Berlin. Moreover, being aware that they were “all sick,” as stated by Mr. Th upon his late departure, they entered a public space with a heightened sensitivity for each other’s hardships. Finally, they were equipped with a new mode of caring for their selves and for others, a capacity they had begun to regain in the therapy sessions in the clinic.

During our boat cruise, we repeatedly crossed the former border between East and West and cruised along specific sites linked to the political history of the separation and reunification of Berlin. We were able to observe the participants’ bodily interactions with each other and in relation to the buildings and objects we passed. They pointed towards specific sites, jointly moving their bodies in the same direction, or moved closer to one another to bend over photos and the information cards in the Vietnamese language we had provided them with. Mr. S’ reaction in view of the White Crosses Memorial for the victims of the Berlin Wall exemplifies a particular enactment of mutual care and resonance. His response was affectively intense as he seemingly relived his experienced injustice. In turning to Mr. Th, his reaction was meant to offend and provoke the latter. Mr. S, who had fled the South of Vietnam, said “The reunification in Germany has been peaceful whereas in Vietnam there was no peace and no justice.” Instead of being offended, Mr. Th, who had been born in the South of Vietnam, too, but had come to the GDR as a contract worker, attempted to empathetically enter the dialog by contextualizing Mr. S’ experience: “Yes, that is true but there was no war in Germany before the reunification, no blood. The long-lasting war in Vietnam had a tremendous impact on how things evolved afterwards.” Mr. S was eventually soothed. In earlier encounters, such emotionally charged behavior would most likely have led to a conflict about what were legitimate reasons for leaving their home country. In this context, however, both made attempts to relationally deal with the situation, i.e. they both applied a mode of responsibility they had been made aware of in the therapy.

The affective resonance differed significantly at the table next to us where the women sat. While bending over the information cards, the women created an immediate sensorial proximity. Mrs. Q, for instance, held a card in the direction of Mrs. L who moved closer and turned toward her to speak about the cards’ content. This, in turn, apparently triggered the women’s readiness to share impressions of the gendered dimensions of the reunification. They joked about the gender imbalance that became visible after the wall had fallen. Whereas a surplus of Vietnamese men lived in the Western part of the city (men often were sent ahead to undertake the dangerous journey out of Vietnam), the women said the situation was reversed in the Eastern part where predominantly Vietnamese women had been hired as contract workers. Mrs. L laughingly explained “When people took first steps to find out about the Vietnamese on the other side of Berlin, a baby boom, of course, followed these encounters.” Compared with the men on the boat, the women obviously more easily gained common ground by means of instantaneous affective contagion, a fact that, we suggest, relates to gendered dimensions of their “emotion repertoires” (von Poser et al. 2019), according to which female care connects to the fostering of socio-emotional harmony.

During the excursion to Berlin’s *Botanical Garden*, we observed how spatiality and sensuality changed bodily composures and consolidated embodied belonging. While walking in a row through a narrow pathway and passing greenish-greyish succulents and plants with thorns, for example, the participants had appeared rather calm, slow-moving, less interactional, and somewhat detached. In telling the others to “better not touch those plants,” Mr. C, for instance, expressed a certain reservation. Upon entering the main tropical greenhouse, by contrast, we noticed an immediate sensual affection among the participants in relation to the warm and humid climate, the scents and smells, and the overtowering plants and trees. Due to their sensual immersion in an environment similar to the one they knew from Vietnam, they instantly started to chat vividly, their facial expressions became more attentive and they took larger and quicker steps toward all kinds of plants. Thus, they stretched their backs and their body postures became relaxed and upright. Interestingly, their bodies also moved closer to one another without adhering to their otherwise internalized gender boundaries between male and female bodies. In pointing to and even touching certain plants, implicit knowledge as well as competence were relationally evoked: the participants vividly exchanged advice on what kinds of palm leaves to use for the bundling of brooms, how to fold things into banana leaves, and which leaves to collect for brewing tea. Mrs. N stood next to Mr. C and they exchanged their memories of a specific plant while touching and smelling the leaves to make sure that they were talking about the same one. Then, she ripped off one leaf and put it into her mouth, and so did Mr. C and eventually Anita, who was involved in that situation. In a subsequent conversation with Edda, Anita reflected on this affectively contagious moment as part of her relational and emplaced fieldwork experience in the following way:

“Mrs. N did not only touch the leaves. She tried the leaves. Yes, yes, yes, and then Mr. C, too, and then, I finally tasted—being aware that I was inside a public park and should not do that. But it was so natural that I tore off the leaf. And also to consume what you are familiar with, not just smell and touch, but incorporate it. I found that exciting.”

Despite being aware of public park regulations, her own senses forcefully had been drawn into this particular embodied situation (cf. Mattes, Kasmani and Dilger 2019). Edda confirmed the vivid and agile mode of interaction between the participants who spontaneously gathered and dispersed again following their sensual impressions. Sharing familiar senses and movements, they created a commonality that was based on deeply embodied memories. We had the impression that through this dense form of immersion the participants were ‘brought out of their shell,’ and we all left the main tropical greenhouse engaged in vibrant conversations. After a picnic at a nearby spot in the shadow of a tree, the day ended with a shared bodily exercise of mindful movements, which the participants knew from their therapy.

The group dynamics during the third excursion to Peacock Island created another series of emplaced affective exchange as an extract from Edda′s fieldnotes illustrates:

Following a path into a forest, (…) Mr. A discovered a peacock and approached the animal slowly. On the island, (…) the animals roam free. He made cautious steps and reached out his hand to touch the peacock. At first, the peacock went in the opposite direction, and Mr. A followed it carefully. The others [i.e. the other participants] closely observed this unusual encounter, stood around with some distance and talked about it with excitement. Mr. A commented on what he did by saying “I try to get closer…” Mr. C, Mr. S, the psychologist, and I pulled out our smartphones to capture the scene. Mr. A kneeled with an outstretched arm and admired the peacock from close. Then, for a short moment, his fingers touched the beak of the bird. Watching this affecting moment connected and captivated us, we all looked curiously at the peacock and Mr. A. Then the peacock turned around and vanished into the bushes. Mr. A stood up, walked towards us, and smiled. The dense affective intensity that captured the observers’ focus on this human-animal encounter suddenly dissolved as Mr. C turned to Mrs. Q and Mr. B and explained how he would prepare a meal of the peacock; he was a trained cook sharing his culinary knowledge. He listed different cooking recipes and preparation steps and contagiously evoked laughter in the others. Mr. C took this as an opportunity to cite more recipes. At that moment, Mr. S appeared next to me and shared yet another impression: he had observed that in Germany all free-roaming animals are so trustful because they knew that nothing would happen to them. In Vietnam, he said, if you saw a free-roaming animal, it would immediately run away because even young children hunt animals to eat them. He said these words with fascination as mirrored in his smile and his wide-opened eyes. (…) Although Mr. C mocked this interaction in the first place, we later observed how he himself approached another peacock while we had a picnic. Like Mr. A, he also assumed a compact body posture and slowly moved towards the peacock with his arm stretched out in front of him.

This rather random situation marked a significant moment of mutual recognition, acceptance, and inclusion. The participants displayed something seemingly simple, which had been overlaid by the pressure related to daily struggles and a deficient self-perception: in this affective community, they joked and laughed, they mocked each other, and with a renewed ease, they attentively resonated with each other. In other words, they solidified their ephemeral community of care.

### Shared Foodways

Apart from shared spatial explorations, we also paid attention to shared foodways. In several ethnographic encounters, both Edda and Anita had learned that the sharing of food matters greatly in socio-emotional ways. In terms of care, it is worthy to note the difference between approaching people in ‘German’ and ‘Vietnamese’ contexts. A common German introductory phrase is ‘How are you?,’ whereas in Vietnamese contexts it is usual to ask ‘Have you already eaten?’ Our first go-along ended with a visit to a traditional Berlin café, a somewhat noisy location underneath an S-Bahn arc, furnished with old carriages and utensils hanging from the ceiling. The participants took seats according to internalized gender roles, which means that women and men sat separated, and we ordered coffee, tea, and cake. Two men kindly refused our invitation to order a cake because “German cakes are too sweet.” In the end, they suggested that next time we should instead eat Phở, a traditional Vietnamese soup. In terms of embodied senses, we found out that the specific spatial and material environment of the café merged with other and obviously discomforting place-memories of some participants. For instance, it was apparent that the location was still reverberating in Mr. B’s sensorium when, in an ensuing encounter in the clinic, he asked us several questions about “German” table manners. He wanted to know about how and where to sit at the table, whether to cross legs and where to place arms, how to hold fork and knife and so on. Asking these seemingly practical questions raised the attention of the other participants who eagerly added further questions. Two aspects became clear: first, due to un-negotiated misunderstandings on the level of direct sensual practices, they felt socially disembedded and displaced within what they considered “*the* German society.” Second, they recognized a commonality in existing felt differences based on estrangement whose long-term affective consequences they had neither noticed nor addressed before.

Compared to this example of socio-emotional discomfort, our second shared culinary go-along evoked a completely different affective resonance. The participants suggested having a picnic in the *Botanical Garden* with everyone contributing something. It turned out to be a culinary medley with potato salad, flavored sticky rice, cake, fruits, and spring rolls. We took off our shoes, sat on blankets, and started to chat in the shadow of some trees. Fruits were cut open and handed around, and plates and bowls went from hand to hand so that everyone could taste the different dishes. Both Mrs. L and Mrs. N had prepared spring rolls. In discussing the ingredients which they had used, they referred back to the different geographies or “sensescapes” (Howes 2006) of Vietnam, where each had grown up. In speaking of different herbs, which either grow in the central highlands or close to the seacoast, they also expressed their relations to different places of birth. The way in which they recognized differences happened in affirmative ways, though. They reciprocally tasted the spring rolls. In so doing, they sensually evoked images of an internally heterogenous, yet basically common home country that neither of them inhabited any longer, an aspect through which they and the others felt united. In previous conversations, during which the participants had remained rather detached in bodily and sensual terms, they had spoken of internal differences in rather disregarding ways. Now, in contrast, the level of mutual disregard was markedly absent.

Our last example of affective contagion triggered through shared foodways, into which Edda was drawn as an emplaced fieldworker, derives from a situation of sensual relatedness that took place at the buffet of a Pan-Asian restaurant. Apart from recommending specific dishes to each other and what kinds of food to combine with what kinds of sauces, we noticed that, while talking, the participants put scoops of rice, fried vegetables, and sushi directly and confidently on each other’s plates. Mrs. L softly embraced Edda and led her to the buffet, where she recommended a specific choice of fried veggies. Some of the other participants entered this culinary dynamic by adding more food on both of their plates, by pointing to other tables where other regional foods were stored and by encouraging to try even more flavors. More than once we were assured that this particular habitualized mode of caring for others via the medium of food was a paramount societal value within the generation of our participants. As mentioned above, the participants had withdrawn from ‘normal’ social life, which included the withdrawal from even basic embodied and relational practices of care. In reviving these practices, at least temporarily, the participants expressed immediate well-being.

### Shared Photos

During our go-along with the participants in the *Botanical Garden*, we also made use of the photovoice method. After explaining the purpose of the exercise, we met after an agreed time to converse about what the participants were drawn to and why. Interestingly, except for Mr. C, who used his photo to speak about the relieving effect of a plant with regard to dry skin, the others all related to their photos by expressing individual and rather ambiguous states of feeling. The following three examples speak to different affective intensities. Showing us two photos he had taken with this smartphone, Mr. S explained:

“The picture depicts a beautiful day, freedom, growth of nature, an ideal nature. The children running around show freedom, they laugh and smile. The second picture makes me sad. Look, on the first one are trees, on the second one are only stones, no trees. That made me think of the war, after a battle every tree was demolished, torn. There were only stones and corpses left. I remembered that while seeing that spot without trees.”^6^

Mr. S’ explanation showed that his perception, while moving through the surroundings, was constantly oscillating between positive and negative moods, associations, and memories. He was able to enjoy the situation that he saw on the first photo but the second photo triggered strong memories of his war experiences. While looking at the first photo, his tone of voice was higher and brighter, whereas he described the second one with a deeper voice and a markedly tense body posture. The reaction of the group resonated with his descriptions; the others smiled as he spoke of the beautiful day, whereas they respectfully turned their gaze down when he spoke of the spot without trees. During this exercise, Mr. S practically became aware of what he had acquired in psychoeducation. By noticing the impact of not only negative but also positive images on his self-perception, he realized that he was not merely passively exposed to his moods, which in the past had made him shut himself off completely, but that he could counter and interrupt his painful memories.

Mr. A shared two photos. First, he spoke about a plant growing in forests, which reminded him of the beautiful plants from a specific forest in Vietnam where he had grown up. The second photo showed a frog and he explained his choice with the following words: “A frog, quak quak. When I was a child, I used to work on the fields and I saw many frogs out there. The frog reminded me of my childhood when I used to live at the river. You could find many animals on the rice paddies.” Mr. A had usually been quite reluctant in thinking of and sharing positive memories relating to his former life in Vietnam. Most of the time he had either avoided talking about Vietnam at all or had only raised criticism. We knew that Mr. A had moved to the GDR twice as he was not able to live up to his own and others’ socio-economic expectations upon his first return to Vietnam. Therefore, on his second departure, he left the country for good. Thus, in consciously distancing himself from his country of birth, he obviously also pushed away memories relating to his childhood, which he now started to draw on as a positive resource. The body in movement, we believe, evoked feelings in him that otherwise had remained unvoiced.

When Mrs. N, the oldest participant in the group, explained her choice of photo in such a low voice that she was nearly inaudible, she was encouraged by Mr. C and Mr. A:

Mrs. N: “I have a picture of people laughing and being happy. The flowers and plants blossom. But beneath, there is a flower that had already bloomed, its head is directed to the ground. The plant is surrounded by happy flowers, but this flower is looking down.”

Mr. A: “That means that flowers are like people. Some people who are happy and others who are sad.”

Mr. C: “The leaves of the flower are hanging down; as if they cry. But it is different with every plant.“

Mrs. N: “It is like with children and grandchildren, they still grow and develop. They are healthy and happy but the old ones, they are unhappy, sad.”

In speaking—almost poetically—of the wilting flower, Mrs. N spoke of her feelings of loneliness, unhappiness, and the end of life. In this exercise, Mrs. N did adhere to an indirect mode of communication she was familiar with in regard to the expression of felt tensions. She did so, however, in a somewhat altered way which shows the dynamics of care relations. According to the participants’ habitualized understandings, conflicts between persons were to be mediated by a third party. In the case of internal conflicts, by contrast, people were expected to either keep their feelings to themselves or to entrust their concerns only to specific social others. One’s own children do not belong to this circle, and neither did the other female participants in the group because they were much younger. Instead, Mrs. N could have approached a *Cô* (younger aunt) or a *Chị* (older sister), but these were not present in Germany or had already passed away. Despite being younger, Mr. A and Mr. C, in their wish to care for Mrs. N in this particular situation, eventually applied a modified sense of shared social sensibility. Normally, men and women would not exchange such personal feelings according to internalized gender roles. Based on a normative understanding of their patriarchal society, however, they felt their male status obliged them to express care for her.

In comparison to the power of shared embodiment that became visible in relation to shared movements and foodways, the photovoice elicitation was certainly less impactful in terms of immediate sensual contagion. After all, it was less emplaced and less relational; the participants went around on their own to take the photos and interpreted the depicted scenes only later in front of the group. Nevertheless, the results of the photovoice exercise made clear that individual afflictions based on both biographical experiences and current positionalities did not completely dissolve in this ephemeral community of care. Importantly, however, the method pointed out the importance of being able to embed individually felt and deeply embodied intensities in a wider dynamic social and spatio-sensorial framework of recognition.

## Discussion

Why did we choose to speak of the affective community that we were able to trace with its consolidating and dissolving moments as a particular ephemeral community of care? Affective communities can have different purposes. During our go-alongs, we gradually discerned that the purpose of this specific affective community was built on the need to express and renegotiate non/belonging and in/exclusion in relation to care. In our understanding, care is a social as well as affective process and always embedded within specific and deeply embodied “emotion repertoires” (von Poser et al. 2019). As argued above, emotion repertoires endow individuals as well as collectives with the agency and security to display, negotiate, and regulate feelings in socio-culturally appropriate ways. More specifically, individuals and collectives internalize these repertoires by means of social and embodied imitation and habituation, thus attributing to them a quality of durability, which is constantly practiced and tested in the relational and interactional affective settings of diverse social and spatial fields; a process whose dynamics the participants learned to recognize in and beyond the clinic.

As Zink argues, “[t]he notion of affective communities draws attention to processes of producing a temporal solidarization between affected and affecting social bodies” (2019:289). As we have shown, the temporal solidarization of our participants formed a prerequisite to reformulate individual perceptions and expectations of care. In our examples, we highlighted how shared spatial explorations and foodways fostered a sense of caring for others by applying an attentive and responsible behavior. This was the case with Mr. Th, who did not react offensively but regulated the potentially provoking comment raised by Mr. S. The interaction with the peacock created another dynamic sphere of immediate sensual affection between the participants as a moment of shared joyful experience. Another example is Mr. A, whose memories of the frog positively reverberated in him and the others. Sharing sensual affections in the tropical greenhouse demonstrated a sudden microrelational form of inclusion; the interaction with plants elicited deeply embedded and embodied knowledge and connected the participants in an immediate, sensorial way. This embodied knowledge allowed the participants to act competently. Later in therapy, it was discussed as a palpable affective resource to draw upon in order to mitigate negative feelings. We further observed an expressed commonality as the participants voiced felt experiences of estrangement and exclusion relating to German table manners, the adherence to which they had not named as an affective effort before. Most importantly, the participants acquired a flexible understanding of care, which became salient when Mr. A and Mr. C transgressed normative gender roles when openly expressing their felt responsibility for Mrs. N. In caring for others in altered contexts, the participants eventually acquired and performed modified relational forms of caring for their selves.

Whereas Zink further suggests that certain affective communities might turn into more durable “emotional communities” (2019:294), whereby she refers to Barbara Rosenwein’s (2006) concept of communities with a belief in strong, compulsory, and indissoluble bonds, this was not the case with our participants’ ephemeral community of care. Rather, the participants’ purpose primarily was to make sense of felt irritations stemming from the various hardships related to their respective biographies and migratory experiences. Once this purpose was achieved, the participants were encouraged to immerse themselves in their emotional communities of everyday life. This process, of course, did not unfold equally for all and is certainly linked to the participants’ individual courses of illness, their modes of medical compliance, their expectations and chances to revitalize social relations, their possibilities to return to work or assume an active role in their family or community as well as to recognize and reduce stressful situations.

The question of who might benefit from such an ephemeral community of care in the long run eventually depends on several and overlapping factors such as one’s social location, one’s occupation, or one’s point of entry into psychotherapy as well as its duration. For example, Mrs. L realized that, in actively caring for others, she could care for herself. After the end of the group therapy, she began to volunteer in supporting elderly people in her community. Ever since, she has been regaining a kind of relational agency, which in turn sustains her well-being. Still, in agreement with her psychotherapist, she needs to remain attentive to not demand too much of herself in caring for others. Mr. A, too, became reintegrated through social engagement in his community in a way in which he could also take recourse to his formerly professionally trained skills. Mr. B decided on further regular psychotherapeutic treatment in another group therapy context. Compared with the other participants, he had had least experience with psychotherapy and was still struggling with his transition back into societial life due to continued unemployment. Mr. S, finally, ended his psychotherapeutic treatment with the end of the group therapy and tried to come to terms with his afflictions by applying what he had learned over the course of the therapy. Whereas before, he had strongly isolated himself from his social surroundings, he now reported that he felt more at ease in involving himself in social interactions. During an encounter with Edda one year later, however, he said that his emotional condition had again worsened. It turned out that he had stopped taking his prescribed medical remedies without consulting his therapist. Nevertheless, he was considering going back to therapy: “I think I am sick again”, he said. “I need to go back to the clinic. What about the group?”

## Conclusion

The aim of this article was to show how a group of elderly Vietnamese migrants, whose self-perceptions were dominated by feelings of numbness, powerlessness, and dependence, came to sensually reenact a mutual and modified form of care as a temporal affective community by playfully “*creating, sustaining and reproducing bodies, selves, and social relationships*” (Nguyen, Zavoretti and Tronto 2017:202; italics in original). Through the notion of affective communities we were able to explore how individual bodies were sensually synchronized without the fear of ‘losing face’ and the pressure to perform prescribed roles and meet social expectations.

We demonstrated how, when, and why bodies resonated within a particular ephemeral community of care. In our case, shared embodiment and emplacement was a way to set in motion processes that revealed implicit and hidden memories and feelings of non/belonging, which functioned as either stressors or resources. Sharing embodied knowledge (re-)established a sense of belonging that the participants practiced within a temporal frame of solidification. Despite their otherwise deficient self-perceptions, over the course of our excursions and through sharing their photos they came to feel accepted as an equal and relational part of an ephemeral community of care. Even though, according to Zink (2019), affective communities may be of short duration, the intensities of feeling that people experience while being part of such an affective community can become positively reverberating embodied memories themselves; this is mirrored, for instance, in Mr. S’ enquiries about the whereabouts of “the group.”

As argued in the beginning of this article, the reactivation of the knowing body in the clinic was a systematic approach to work with bodily engrained memories and intensities. Systematic phenomenological go-alongs outside the clinic proved a valuable and, indeed, necessary supplement to clinical psychotherapeutic treatment. Not only did they help to trace hidden knowledge but also, to become durable tools of meaning-making, cognitive learning processes in general have to merge with processes of embodying and habitualizing cognitions. Phenomenological go-alongs, we believe, are one way to foster these processes in more random situations of everyday life-worlds.

Coming back to the particularities in our case, it is important to keep in mind that the participants had lacked words and voices to express feelings of estrangement and discomfort in relation to migration, on the one hand, and were not able to make sense of this lack, on the other. Therefore, their bodies had to be ‘asked’ and their senses had to be synchronized. Our anthropological approach of ‘asking bodies and synchronizing senses’ was based on systematic phenomenological go-alongs outside the clinic during which we paid specific attention to our participants’ immediate sensorial perceptions and affective appropriations of spatio-social worlds. We noticed that the participants’ modes of being differed markedly when they actually moved through and interacted with social, material, and spatial enviroments compared to when they just talked about such interactions in the clinical setting. They were able to bring out and share implicit and hidden stresses and strains in concrete situations of affecting and being affected by others. Later, in the therapy, they eventually addressed and contextualized these hidden stresses and strains. As a result, a shared sensual space unfolded in which words or phrases such as “we are all sick” eventually could become a starting point to express and negotiate what was commonly felt.

To conclude, we hope to have conveyed the idea that (psycho-)therapeutic treatments in a world in motion could benefit from methodologies in motion such as the phenomenological one that we pursued with a group of elderly Vietnamese migrants in Berlin. Through an emplaced exploration of shared sensations and spatiality we were able to reveal some of the health-related and deeply embodied ruptures in regard to people’s feelings of non/belonging and in/exclusion that often remain unaddressed in current politics of belonging and health.

## Footnotes

1 Our ethnographic research was conducted within an anthropological-psychiatric research project (A02). The project is part of the Collaborative Research Center 1171 “Affective Societies—Dynamics of Social Coexistence in Mobile Worlds” based at Freie Universität Berlin and funded by the German Research Foundation (DFG). We would like to thank our psychiatric and psychological colleagues Thị Minh Tâm Tạ, Eric Hahn and Main Hương Nguyễn for an inspiring interdisciplinary collaboration.

2 The names of our protagonists are anonymized throughout the article.

3 Whereas we use the term “emotions” to refer to socio-culturally, historically, and politically shaped feelings (von Scheve and Slaby 2019), the term “affect” denotes the relationally occurring intensities in “encounters between bodies that involve a change—either enhancement or diminishment—in their respective bodily *capacities* or micro-powers” (Slaby and Mühlhoff 2019:27).

4 We call the places of these encounters “affective niches” in accordance with Colombetti, Krueger and Roberts’ *Affectivity Beyond the Skin* approach which, rather than focusing on internal individual states of brains and bodies, explores affectivity as “scaffolded by resources beyond the head” (2018:1). Affective niches are all kinds of (self-styled) environmental settings “providing the developmental conditions for affective states to take shape and thrive” (ibid).

5 The meditation practice of “mindful movements” is an embodied exercise for well-being that goes back to the teachings of Thích Nhất Hạnh, a Vietnamese buddhist monk and zen master. In 1982, he established the buddhist Mindfulness Practice Center Plum Village in Southern France (https://plumvillage.org/). Prior to the beginning of the cognitive behavior group therapy with a focus on affects and emotions as led by our psy-colleagues, our anthropological–psychiatric team visited Plum Village in July 2016.

6 Recorded descriptions given to us in Vietnamese were translated into English by our colleague Phương Thúy Nguyễn. We thank her very much for her engagement.

## References

[CR1] Behrens, Julia, and Bùi Kim Đĩnh 2019. “No War No Vietnam: A Multimedia Art Exhibit in Berlin.” *Journal of Vietnamese Studies* 14: 87-96.

[CR2] Colombetti, Giovanna, Joel Krueger, and Tom Roberts 2018. “Editorial: Affectivity Beyond the Skin.” *Frontiers in Psychology* 9 (1307): 1-2. 10.3389/fpsyg.2018.01307 PMC6068265 30090083

[CR3] Desjarlais, Robert, and C. Jason Throop 2011. “Phenomenological Approaches in Anthropology.” *Annual Review Of Anthropologys* 40: 87-102.

[CR4] Elias, Norbert 2000. *The Civilizing Process*. Malden: Blackwell.

[CR5] Glick Schiller, Nina, and Noel B. Salazar 2013. “Regimes of Mobility Across the Globe.” *Journal of Ethnic and Migration Studies* 39: 183-200.

[CR6] Heyken, Edda, Anita von Poser, Eric Hahn, Thi Main Huong Nguyen, Jörg-Christian Lanca, and Thi Minh Tam Ta 2019. “Researching Affects in the Clinic and Beyond. Multi-perspectivity, Ethnography, and Mental Health-Care Intervention.” In *Analyzing Affective Societies. Methods and Methodologies*, edited by Antje Kahl, 249-264. New York: Routledge.

[CR7] Howes, David 2006. *Empire of the Senses: The Sensual Culture Reader.* Oxford: Berg Publishers.

[CR8] Kusenbach, Margarethe 2003. “Street Phenomenology: The Go-along as Ethnographic Research Tool.” *Ethnography* 4: 455-85.

[CR9] Mattes, Dominik, Omar Kasmani, Marion Acker, and Edda Heyken 2019. “Belonging.” In *Affective Societies: Key Concepts*, edited by Jan Slaby and Christian von Scheve, 300–309. London, New York: Routledge.

[CR10] Mattes, Dominik, Omar Kasmani und Hansjörg Dilger 2019. “‘All Eyes Closed’. Dis/sensing in Comparative Fieldwork on Affective-religious Experiences.” In *Analyzing Affective Societies. Methods and Methodologies*, edited by Antje Kahl, 265–278. London, New York: Routledge.

[CR11] Nguyen, Main Huong, Jörg-Christian Lanca, Eric Hahn, Anita von Poser, Edda Heyken, Katja Wingenfeld, Roland Burian, Albert Diefenbacher, and Thi Minh Tam Ta in print. “Perceived Emotional Distress among Vietnamese Outpatients in Germany. An Interdisciplinary, Mixed-method Study.” *Transcultural Psychiatry*. 10.1177/1363461520920329 32389070

[CR12] Nguyen, Minh T. N., Roberta Zavoretti, and Joan Claire Tronto 2017. “Beyond the Global Care Chain: Boundaries, Institutions and Ethics of Care.” *Ethics and Social Welfare* (11) 3: 199-212.

[CR13] Pink, Sarah 2015. *Doing Sensory Ethnography*. 2nd Edition. Los Angeles: SAGE.

[CR18] Röttger-Rössler, Birgitt 2018. “Multiple Belongings. On the Affective Dimensions of Migration”. *Zeitschrift für Ethnologie* 143: 237-262.

[CR19] Röttger-Rössler, Birgitt, Gabriel Scheidecker, and Anh Thu Anne Lam 2019. “Narrating Visualized Feelings: Photovoice as a Tool for Researching Affects and Emotions among School Students.” In *Affective Societies: Methods and Methodologies*, edited by Antje Kahl, pp. 78-97. London, New York: Routledge.

[CR20] Rosenwein, Barbara H. 2006. *Emotional Communities in the Early Middle Ages*. Ithaca, NY: Cornell University Press.

[CR21] Sevenhuijsen, Selma 2003. “The Place of Care: The Relevance of the Feminist Ethic of Care for Social Policy.” *Feminist Theory* 4 (2): 179–197.

[CR22] Slaby, Jan, and Rainer Mühlhoff 2019. “Affect.” In *Affective Societies: Key Concepts,* edited by Jan Slaby and Christian von Scheve, pp. 27-41. London, New York: Routledge.

[CR23] Tronto, Joan Claire 1993. *Moral Boundaries: A Political Argument for an Ethic of Care.* New York: Routledge.

[CR14] von Poser, Anita 2017. “Care as Process. A Life-course Perspective on the Remaking of Ethics and Values of Care in Daiden, Papua New Guinea.“ *Ethics and Social Welfare* 11(3): 213-229.

[CR15] von Poser, Anita 2018. “*Affective Lives* im Vietnamesischen Berlin: Eine emotionsanthropologische Perspektive auf Zugehörigkeiten, Alter(n) und (Im-)Mobilität.” *Geschichte und Gesellschaft. Zeitschrift für historische Sozialwissenschaft* 44 (2): 285-311.

[CR16] von Poser, Anita, Edda Heyken, Thi Minh Tam Ta, and Eric Hahn 2019. “Emotion Repertoires.” In *Affective Societies. Key Concepts,* edited by Jan Slaby and Christian von Scheve, pp. 241-251. London, New York: Routledge.

[CR17] von Scheve, Christian and Jan Slaby 2019. “Emotion, emotion concept”. In *Affective Societies. Key Concepts,* edited by Jan Slaby and Christian von Scheve, pp. 42–51. London, New York: Routledge.

[CR24] Zink, Veronika 2019. “Affective Communities.” In *Affective Societies: Key Concepts*, edited by Jan Slaby and Christian von Scheve, pp. 289-299. London, New York: Routledge.

